# Microbial Communities in the Extraradicular and Intraradicular Infections Associated With Persistent Apical Periodontitis

**DOI:** 10.3389/fcimb.2021.798367

**Published:** 2022-01-12

**Authors:** Xiaoqiang Sun, Zi Yang, Yong Nie, Benxiang Hou

**Affiliations:** ^1^ The Department of Endodontics, School of Stomatology, Capital Medical University, Beijing, China; ^2^ College of Engineering, Peking University, Beijing, China

**Keywords:** microbial community, persistent apical periodontitis, root canal, periapical lesions, 16S rRNA sequencing, sinus tract

## Abstract

Microorganisms in the complex root canal system and the extraradicular regions, including the periapical lesions and extraradicular biofilm may cause root canal treatment failures. However, few studies described the difference between the intraradicular and extraradicular infections from the same tooth associated with persistent apical periodontitis. This study aimed to characterize the microbiome present in the root canal, extraradicular biofilm, and periapical lesions associated with persistent apical periodontitis. The microbial communities in the root canal, extraradicular biofilm, and periapical lesions were investigated by Illumina high-throughput sequencing using Illumina Hiseq 2500 platform. The dominant phyla in the extraradicular and intraradicular infections associated with persistent apical periodontitis were *Proteobacteria, Firmicutes, Bacteroidetes*, and *Actinobacteria*, and the genera *Fusobacterium*, *Morganella, Porphyromonas, Streptococcus*, and *Bifidobacterium* dominated across all samples. Although extraradicular infection sites showed higher OTU richness and β-diversity compared to intraradicular samples, the occurrence of sinus tract rather than the sampling sites demarcated the microbial communities in the infections associated with persistent apical periodontitis. PERMANOVA analysis confirmed that the samples with or without sinus tracts contained significantly different microbial communities. *Porphyromonas, Eubacterium, Treponema*, and *Phocaeicola* were found in significantly higher levels with sinus tracts, whilst *Microbacterium* and *Enterococcus* were more abundant in samples without sinus tracts. In conclusion, diverse bacteria were detected in both intraradicular and extraradicular infections associated with persistent apical periodontitis, which might be influenced by the occurrence of the sinus tract. The results may provide new insight into the pathogenesis of persistent apical periodontitis.

## Introduction

Persistent apical periodontitis has been reported to occur in 10-20% of teeth, even when the root canals are thoroughly prepared, disinfected, and obturated, leading to periapical lesions persist after the failure of endodontic treatment and retreatment (Nair, 2004). Residual bacterial biofilm located in the complex apical root canal system and extraradicular regions was considered to be an important factor affecting the persistent apical infections (Zakaria et al., 2015; Bronzato et al., 2021).

By using transmission electron microscopy, complex multispecies biofilm was present in root canals of persistent apical periodontitis (Carr et al., 2009). *Fusobacterium*, *Corynebacterium, Porphyromonas, Streptococcus*, and *Stenotrophomonas* were the most abundant genera detected in intraradicular samples of persistent infection (Henriques et al., 2016; Takahama et al., 2018; Gomes et al., 2021; Hou et al., 2021). Moreover, *Pseudomonas* spp., *Burkholderia* spp., and *Enterococcus faecalis* were also found highly prevalent in root canals with persistent endodontic infections (Siqueira et al., 2011; Barbosa-Ribeiro et al., 2021).

Using scanning electron microscopy, extraradicular biofilm was observed on the surface of cementum from root tip to coronal (Wang et al., 2012), and was detected in 80-100% of root canal treatments considered endodontic failure (de Sousa et al., 2017). Meanwhile, the biofilm attached to the external surface of the root could not be easily controlled by conventional root canal preparation and disinfection (Ping et al., 2015). Thus, the intraradicular and extraradicular regions in the core apical area play an essential role in the persistent chronic inflammatory process.

The difference in the bacterial composition of periradicular lesions and root ends (microbiota inside and outside of the root canals) was characterized by 16S rRNA cloning and sequencing (Subramanian and Mickel, 2009). Ten crucial microorganisms from root ends and periapical lesions were quantified by real-time PCR (Pereira et al., 2017). The mass spectrometry was used to compare bacterial and human metaproteome collected from the root apexes and matched apical lesions of persistent infections (Provenzano et al., 2016). And we have also compared the microbial profiles between periapical lesions and extraradicular biofilm by ribosomal 16S rRNA cloning and sequencing in our previous work (Zhang et al., 2021). However, few studies described the difference between the intraradicular and matched extraradicular infections associated with persistent apical periodontitis, and the facilitating factors of persistent extraradicular infection were still unknown.

Microbial diversity in samples has been traditionally explored by culture methods involving isolation and identification, and, afterward, by molecular technology. Recently, with the development of molecular technologies, next-generation high-throughput sequencing techniques provide a more thorough understanding of oral microbial communities (Gomes et al., 2015; Zandi et al., 2018; Hou et al., 2021). Microbial profiles of persistent periapical lesions were characterized by 454-pyrosequencing technology and were of greater bacterial diversity than previous traditional approaches (Saber et al., 2012). Compared to 454 pyrosequencing, Illumina sequencing has greater output and lower cost, and it is more accurate because of its lower rate of sequencing errors, with the disadvantages that are relatively short read length and long run time (Di Bella et al., 2013).

To the best of our knowledge, the comparative analysis of microbial profiles in the root canal and its matched extraradicular infections after conventional endodontic treatment have not been studied. Thus, the present study aimed to evaluate the bacterial communities of intraradicular root canal fillings, extraradicular biofilm, and the matched inflammatory lesions from teeth with persistent apical periodontitis by using a high-throughput Illumina sequencing.

## Methods and Materials

### Patient Population and Clinical Examination

Protocols for all procedures were approved by the Ethics Committee of the Dental School of the Capital Medical University, Beijing, China [KJ-2018-018-C-02-FS(CS)], and all patients signed their informed consent form for their participation in this study.

In all, ten samples were collected from eight patients from 21 to 51 years of age with persistent apical periodontitis referred for endodontic surgery were recruited. All selected teeth had previously received root canal treatment and retreatment more than 1 year earlier and exhibited periapical radiolucent areas with satisfactory root canal obturation and restoration. All teeth were examined by X-ray and CBCT to determine the root canal obturation. The coronal restoration was examined before surgery, and after the raise of the mucoperiosteal flap, the edge of coronal restoration was examined in surgery under high magnification. All teeth were symptomatic and the clinical/radiographic features were found and recorded. Five of the ten teeth had a preoperative sinus tract. Teeth presenting with periodontal pockets >3mm, root fractures, separated endodontic instruments, root canal deviations, perforations, and patients treated with antibiotics within 3 months were excluded from the study.

### Sample Collection

**Figure 1:**
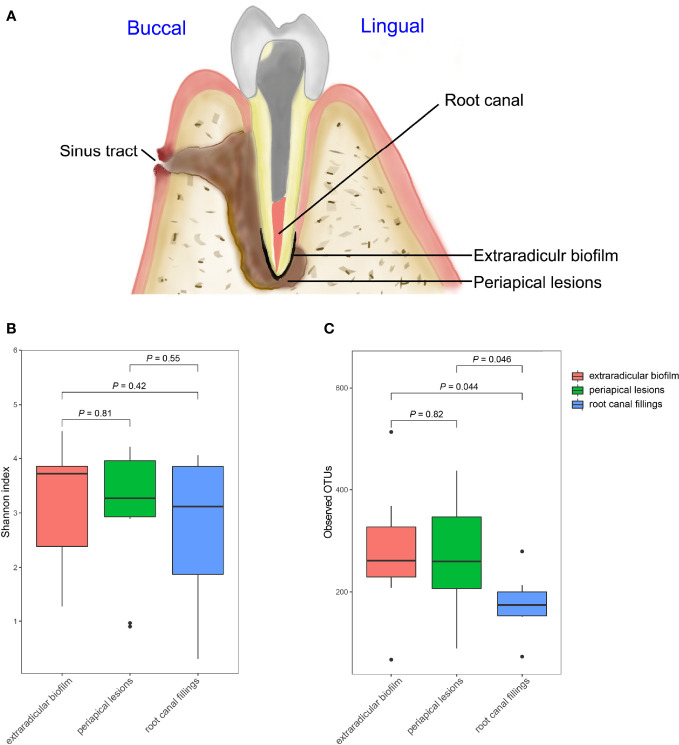
Comparisons of Alpha diversities of samples collected from the root canal, extraradicular biofilm, and periapical lesions. **(A)** schematic diagram of sampling; **(B)** comparison of Shannon index; **(C)** Comparison of observed OTUs. The *P*-values were calculated using the student’s t-test.

All samples were taken from the root canal fillings, root end surface, and matched periapical soft lesions according to the following protocol (**Figure 1A**). All patients were treated by the same surgeon who is an endodontic specialist under high magnification with a dental operating microscope (Leica M525 F40, Germany).

First, patients rinsed with 0.12% chlorhexidine for mouthwash. After applying local anesthesia, the area to be operated on was washed thoroughly by using sterile gauze soaked in 70% alcohol to avoid external contamination. After crevicular and intrasulcular incision, a full-thickness mucoperiosteal flap was lifted. Care was taken to avoid contamination of the surgical site with saliva. After the reflection of the flap, periosteal tissue samples were collected from areas adjacent to the surgical site using curettes and absorbent paper cones to test for bacterial contamination. The apical periodontitis lesions were enucleated by a sterile curette and placed in a sterile Eppendorf (EP) tube containing phosphate buffered solution (PBS) and then immediately frozen in a -20°C refrigerator. The apical 3 mm of the root apexes were washed with sterile saline to remove the blood and planktonic bacteria. Then extraradicular biofilm specimens were taken to curettage the surface of cementum within 2 to 3 mm from root tip to coronal, and immediately placed in EP tubes containing sterile PBS and then frozen in -20°C refrigerator. Then resected to the long axis of the tooth in 2mm. The root canal fillings were curetted and immediately placed in EP tubes containing sterile PBS and then frozen in -20°C refrigerator. Surgery was completed by retrograde preparation and bioceramic material was used for the retrograde obturation.

### DNA Extraction and 16S rRNA Gene Sequencing

DNA of all samples was extracted and purified according to the protocol of the QIAamp DNA Mini Kit (Qiagen, Hilden, Germany) following the manufactural instructions and then was stored at -20°C. To evaluate the microbial composition and diversity per sample, Illumina Hiseq 2500 sequencing (Novogene Bioinformatics Technology Co, Ltd) was performed, according to the manufacturers’ instructions. All the DNA samples should satisfy the quality and quantity standards of high-through sequencing. PCR reactions used the special primers, 515F (5′, GTGCCAGCMGCCGCGGTAA, 3′) and 806R (5′, GGACTACNNGGGTATCTAAT, 3′), which target the V3-V4 hypervariable regions of 16S rRNA genes. PCR amplification was performed in 30 µL reactions containing 15 µL of Phusion^®^ High-Fidelity PCR Master Mix(New England Biolabs, USA) with GC Buffer, 0.2 µM of 515F primer, 0.2 µM of 806R primer, and approximately 10 ng template DNA. The temperature conditions were initial denaturation at 98°C for 1 min, followed by 30 cycles at 98°C for 10 s, annealing at 50°C for 30 s, and elongation at 72°C for 30 s. Finally, 72°C for 5 min.

All PCR products were run and confirmed by 2% agarose gel electrophoresis. Using QuantiFluor™-ST Fluorometer (Promega, China), samples with the bright main strip between 400 and 450 bp were chosen for further experiments. Sequencing libraries were generated using TruSeq™ DNA Sample Prep Kit (Illumina, USA) according to the manufacturer’s introductions and index codes were added. The library quality was assessed on the Qubit R 2.0 Fluorometer (Thermo Fisher Scientific, USA) and Agilent Bioanalyzer 2100 system (Agilent, USA). At last, libraries for each of all samples were sequenced on an Illumina HiSeq 2500. The raw reads were deposited into the National Microbiology Data Center (NMDC) with the accession number: NMDC40013660.

### Bioinformatic Analysis, Statistical Analysis, and Visualization

First, the paired-ended raw reads obtained from sequencing were quality-filtered using Trimmomatic (Bolger et al., 2014). Then the filtered reads were further processed using the Quantitative Insights into Microbial Ecology (QIIME) bioinformatics pipeline (Caporaso et al., 2010). Sequences with ≥ 97% similarity were assigned to the same OTUs. A representative sequence was picked for each OTU and the taxonomic information for each representative sequence were assigned against SILVA ribosomal RNA database and Human Oral Microbiome Database (HOMD) (Chen et al., 2010). Sequences identified as mitochondrial or of chloroplast origin and singleton OTUs were discarded. Shannon indexes were calculated for the evaluation of alpha diversity among different sites. The student’s t-test and Wilcoxon rank-sum test were used for statistical analysis. To compare the microbial profiles of different sites and different symptoms, principal coordinate analysis (PCoA) based on the bray-curtis distances and permutational multivariate analysis of variance (PERMANOVA) were performed. Random forest analysis was used to test whether the bacterial communities of samples with or without sinus tract could be classified by some genera.

## Results

### General Information From 16S rRNA Gene High-Throughput Sequencing

To investigate the microbial communities associated with persistent apical periodontitis, microbial samples from root canal, extraradicular biofilm and periapical lesions from patients with persistent apical periodontitis were investigated by 16S rRNA gene high-throughput sequencing. No bacterial DNA was detected from samples of periosteal tissue, confirming the sterility of the sampling approach. Bacteria were detected in all periapical lesion samples, 9 of 10 root canal and 9 of 11 extraradicular biofilm samples by PCR amplification with the universal 16S rRNA gene primers. A total of 1317195 16S rRNA gene V3-V4 fragments and 1467 OTUs at 0.03 distances were obtained from all the samples (**Table 1**).

**Table 1:** Sample information.

Patient Information			Sample information				General sequencing information		
Patient	Gender	Age	Tooth ID	Sinus Tract	Sample ID	Sampling Site	Number of sequences	Observed OTUs	Shannon index
1	Male	40	1	Yes	A2	root canal fillings	55360	173	3.12
					A3	extraradicular biofilm	31625	241	3.50
					A4	periapical lesions	39316	241	3.48
			2	Yes	B2	root canal fillings	35541	150	3.85
					B4	extraradicular biofilm	42688	281	3.85
					B3	periapical lesions	35016	277	2.89
2	Female	29	3	No	C1	root canal fillings	45236	213	4.06
					C2	extraradicular biofilm	32855	368	3.73
					C5	periapical lesions	44039	437	4.22
					C3	periapical lesions	44751	390	4.08
			4	No	C7	root canal fillings	45498	279	3.86
					C4	extraradicular biofilm	44146	327	3.72
					C8	periapical lesions	35702	361	4.18
3	Female	45	5	No	D4	root canal fillings	73876	152	3.42
					D1	extraradicular biofilm	50636	514	4.51
					D6	periapical lesions	71284	303	3.60
4	Female	32	6	Yes	D15	extraradicular biofilm	48239	261	3.93
5	Male	47	7	No	D8	root canal fillings	34411	175	0.99
					D3	extraradicular biofilm	48212	66	1.27
					D5	periapical lesions	81702	88	0.97
6	Female	51	8	Yes	D13	root canal fillings	54876	200	2.69
					D10	periapical lesions	30569	242	3.06
7	Male	29	9	Yes	GN	root canal fillings	50432	167	1.87
					GY	extraradicular biofilm	70119	208	1.64
					RY	periapical lesions	66428	114	3.05
8	Male	32	10	No	D11	root canal fillings	39711	72	0.31
					D14	extraradicular biofilm	33234	229	2.38
					D7	periapical lesions	31693	195	0.90

### Diversity of Bacterial Communities in Root Canal, Extraradicular Biofilm and Periapical Lesions

**Figure 2:**
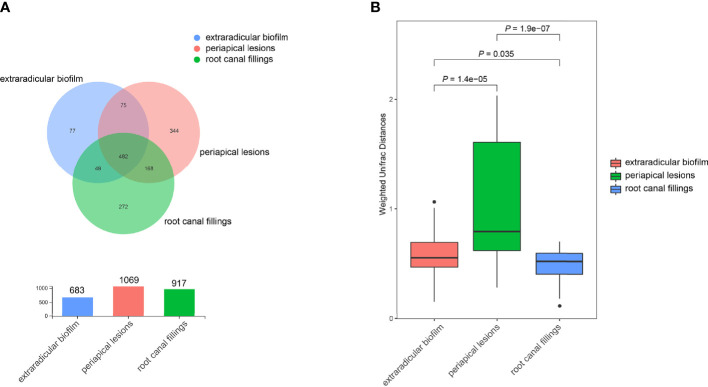
Distribution of OTUs in samples collected from the root canal, extraradicular biofilm, and periapical lesions. **(A)** the Venn diagram indicates the shared/unique OTUs in samples from the root canal, extraradicular biofilm, and periapical lesions; **(B)** comparison of beta-diversities between samples within each group. The *P*-values were calculated using the student’s t-test.

The OTU richness of all samples ranged from 66 to 514, and the Shannon index ranged from 0.31 to 4.51. The mean number of OTUs per sample was 175.7 ± 55.6 in the intraradicular infection group, 277.2 ± 122.5 in the extraradicular biofilm group, and 264.8 ± 113.6 in the periapical lesion group, respectively (**Table 1**). Although the Shannon indices from all three sampling sites were similar (**Figure 1B**), samples from the extraradicular infection sites (i.e., periapical lesion samples and extraradicular biofilm samples) exhibited significantly higher OTU richness than the samples from intraradicular infection site (i.e., root canal filling samples) (**Figure 1C**). Moreover, the number of total OTUs detected in both the extraradicular biofilm and periapical lesions were higher than the root canal filling samples (i.e., 1069 OTUs and 971 OTUs vs. 683 OTUs), which indicated the microbial compositions in the extraradicular infection sites were more variable than the intraradicular infection site (**Figure 2A**). The further β-diversity analysis also showed that the weighted unifrac distances between samples within both the extraradicular biofilm and the periapical lesions groups were significantly higher than those in the root canal fillings (**Figure 2B**).

We also tested the effects of the sinus tracts on the diversity of microbial communities in intraradicular and extraradicular infections, the samples with or without sinus tracts exhibited similar alpha diversities, which suggested that the sinus tracts had weak effects on the microbial richness of intraradicular and extraradicular infections.

### Composition of Bacterial Communities in Root Canal, Extraradicular Biofilm and Periapical Lesions

**Figure 3:**
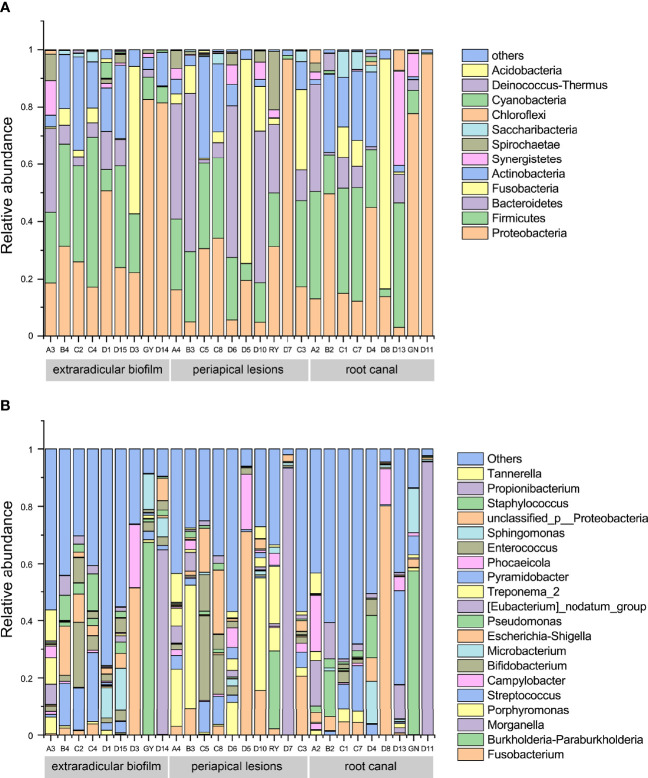
Bacterial compositions of samples from the root canal, extraradicular biofilm, and periapical lesions. **(A)** bacterial compositions at the phylum level; **(B)** bacterial compositions at the genus level.

To investigate the compositions of bacterial communities associated with persistent apical periodontitis, the representative sequences of each OTU were classified against the reference sequences using the Silva classifier (Release138 http://www.arb-silva.de). All the OTUs were assigned to 31 bacterial phyla and 557 genera. Although, the results showed that the bacterial compositions varied among individuals, *Proteobacteria, Firmicutes, Bacteroidetes*, and *Actinobacteria* were detected in all samples and identified as dominant phyla across all the samples, with a mean relative abundance of. 31.5%, 20.9%, 13.2% and 10.5%, respectively (**Figure 3A**). The results suggested that these four phyla represented the core microbiome of extraradicular and intraradicular infections associated with persistent apical periodontitis. It was notable that *Fusobacteria* was also found in most of the samples with a mean relative abundance of 10.4%, however, its relative abundance highly varied across all samples. At the genus level, *Fusobacterium* (with a mean relative abundance of 9.8%), *Morganella* (9.1%), *Burkholderia* (5.4%), *Porphyromonas* (5.3%), *Streptococcus* (4.8%), and *Bifidobacterium* (3.7%) dominated across all samples (**Figure 3B**).

We also annotated the OTUs against the Human Oral Microbiome Database (HOMD). The results showed that the dominant OTUs across all samples presented the highest similarities with strains from *Burkholderia*, *Fusobacterium*, *Proteus*, *Bifidobacterium*, *Streptococcus*, *Sphingomonas*, and *Porphyromonas* (**Table 2**). Most of the abundant genera based on both Silva and HOMD databases were similar, except for *Morganella* from the Silva database and *Proteus* from HOMD. It is notable that although the most abundant OTU showed the highest similarity with *Burkholderia cepacia* HMT-571 Strain: ATCC 25416 from the HOMD database, the sequence identity was only 94.92%, which suggested that it was novel to the HOMD database.

**Table 2:** Top OTUs classified against the HOMD database.

Rank	OTU ID	Sum of OTUs in all samples	HOMD	Identity (%)
1	OTU1570	169504	*Burkholderia cepacia* HMT-571 Strain: ATCC 25416	94.923
2	OTU1470	132710	*Fusobacterium nucleatum* subsp. animalis HMT-420 Strain: NCTC 12276	99.065
3	OTU983	79496	*Proteus mirabilis* HMT-676 Strain: ATCC 29906	95.333
4	OTU805	76149	*Bifidobacterium breve* HMT-889 Strain: ATCC 15700	99.08
5	OTU1567	62369	*Streptococcus oralis* subsp*. dentisani clade 058* HMT-058 Clone: BW009	99.333
6	OTU1593	35142	*Sphingomonas echinoides* HMT-003 Clone: nby481c06c1	98.824
7	OTU1480	31288	*Porphyromonas gingivalis* HMT-619 Strain: DSM 20709	99.326
8	OTU1402	30554	*Campylobacter gracilis* HMT-623 Strain: ATCC 33236	99.061
9	OTU1562	30112	*Peptostreptococcaceae [XI][G-6] [Eubacterium] nodatum* HMT-694 Strain: ATCC 33099	98.824
10	OTU365	30049	*Escherichia coli* HMT-574 Strain: not listed	99.333
11	OTU363	28506	*Enterococcus faecalis* HMT-604 Strain: not listed	99.333
12	OTU1435	27434	*Microbacterium flavescens* HMT-186 Strain: C24KA	98.605
13	OTU10	26143	*Bacteroidaceae* [G-1] bacterium HMT 272 Clone: _X083	99.324
14	OTU996	23485	*Pyramidobacter piscolens* HMT-357 Strain: W5455	99.532
15	OTU113	22976	*Porphyromonas endodontalis* HMT-273 Strain: ATCC 35406	99.327
16	OTU44	21569	*Tannerella forsythia* HMT-613 Strain: FDC 338	98.876
17	OTU234	21493	*Bacillus subtilis* HMT-468 Strain: ATCC 6633	99.113
18	OTU1427	21433	*Pseudomonas stutzeri* HMT-477 Strain: KC	98.222
19	OTU7	17587	*Desulfovibrio* sp. HMT 040 Clone: BB161	96.222
20	OTU126	16728	*Cutibacterium acnes* HMT-530 Strain: JCM 6425	99.302

### The Sinus Tracts Influence the Microbial Community of Extraradicular and Intraradicular Infections

**Figure 4:**
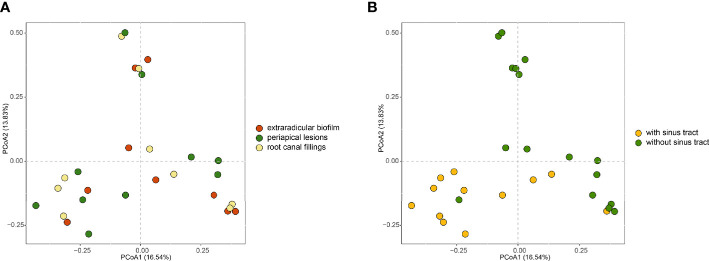
PCoA plots of samples in intraradicular and extraradicular infections associated with persistent apical periodontitis. **(A)** PCoA plot of samples grouped based on the sampling sites; **(B)** PCoA plot of samples grouped based on the existence of sinus tract.

To investigate how the microbial communities of the extraradicular and intraradicular infections associated with persistent apical periodontitis were determined, we compared the microbial communities of samples from different sites and with or without sinus tracts. Principal coordinate analysis (PCoA) based on the bray-curtis distances showed that intraradicular infection, extraradicular biofilm, and periapical lesion samples had similar microbiota profiles (**Figure 4A**). However, the principle coordinates demarcated samples with sinus tract from those without sinus tract (**Figure 4B**). Further PERMANOVA analysis confirmed that the samples with or without sinus tracts contained significantly different microbial communities (PERMANOVA analysis, *R* ^2^ = 0.1, *P* = 0.001). The results suggested that bacterial communities in intraradicular and extraradicular infections associated with persistent apical periodontitis were determined by the sinus tracts.

**Figure 5:**
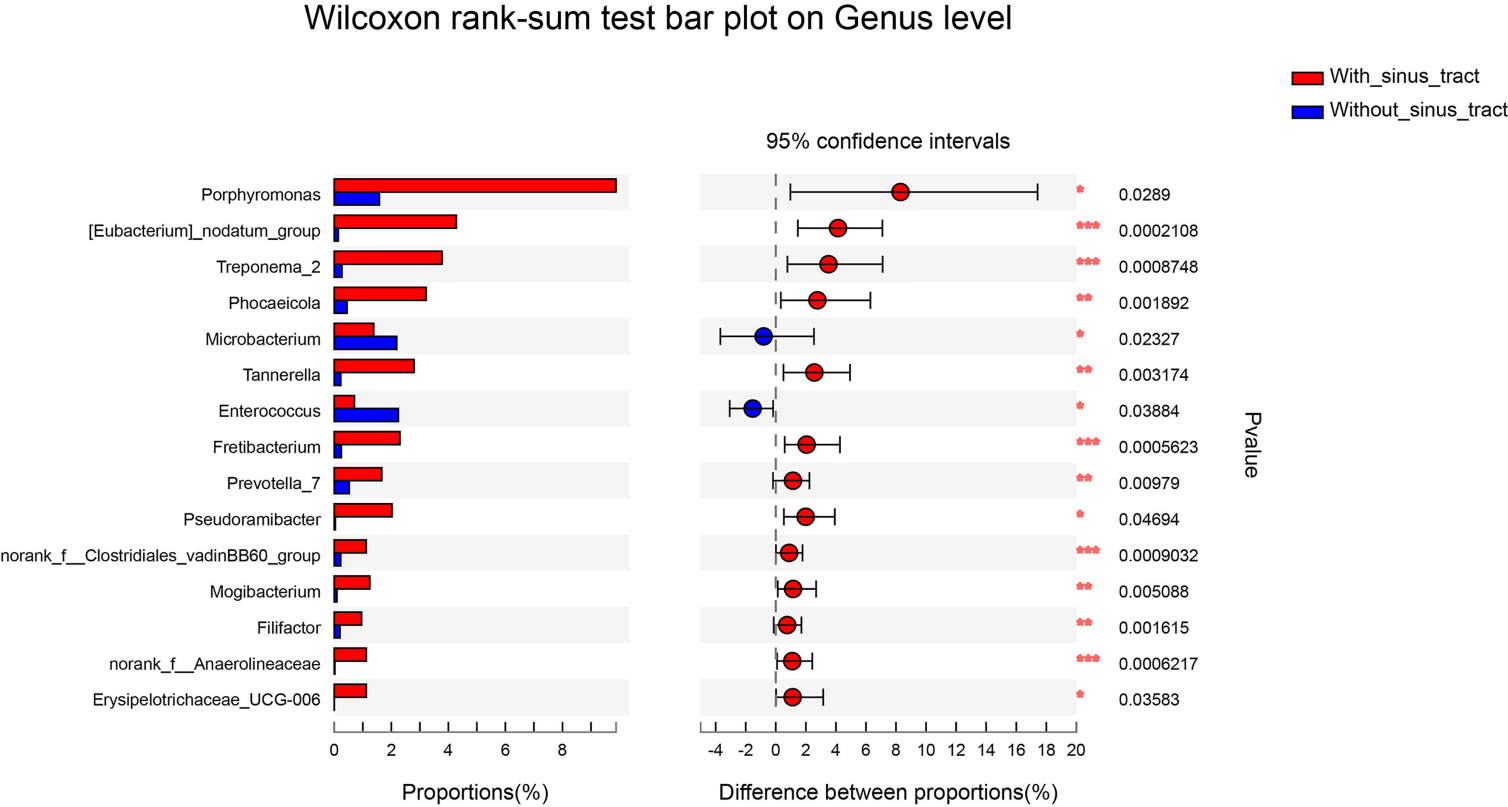
Genera presented significantly different relative abundances between samples with or without sinus tracts. *P < 0.05; **P < 0.01; ***P < 0.001.

**Figure 6:**
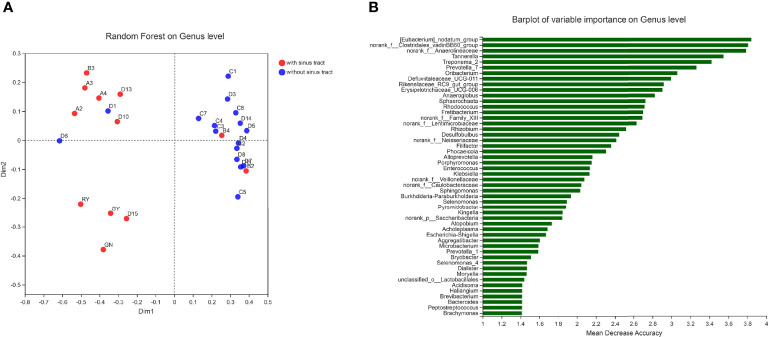
Classification of samples by Random forest analysis. **(A)** sample distribution in the Random forest model; **(B)** the genera with the highest importance scores in the Random forest model.

To assess which genera were most associated with the infections with and without the sinus tracts, we compared the microbial compositions of samples from the two groups. The results showed that *Porphyromonas*, *Eubacterium*, *Treponema*, *Phocaeicola*, etc. were significantly enriched in the samples with sinus tracts. The samples without sinus tracts were characterized by *Microbacterium* and *Enterococcus* (**Figure 5**). We then performed Random forest analysis to test whether the bacterial communities of samples with and without sinus tract could be classified by some genera. The results showed that the Random forest model could classify most of the samples into correct groups (**Figure 6A**), which suggested that there existed consistent differentiation in microbial communities of samples with or without sinus tract. The most important genera for classification included *Eubacterium*, *Tannerella*, *Treponema*, *Prevotella*, and *Oribacterium* (**Figure 6B**).

## Discussion

It is universally acknowledged that microbial communities play an important role in the etiology of persistent infections (Siqueira and Rôças, 2009). Traditionally it has been held that the microorganisms in the necrotic tissues of the root canal systems and periapical tissues could form biofilm on surfaces of root ends. Many studies addressed the microbiota in root ends and extraradicular infection up to now (Subramanian and Mickel, 2009; Zakaria et al., 2015), but the relation and difference among the intraradicular microbiome in the root canal system, extraradicular biofilm, and the matched extraradicular lesions of persistent apical periodontitis were still unclear.

Recently, lots of culture-independent methods were used to investigate the diversity of bacterial microbiome associated with persistent periapical infections, such as Sanger sequencing (Zakaria et al., 2015), microarrays (Qi et al., 2016), and next-generation sequencing (Saber et al., 2012). The diversity of oral flora detected by high-throughput sequencing is one to two orders of magnitude higher than other methods (Keijser et al., 2008; Di Bella et al., 2013). The high-throughput Illumina sequencing facilitated the acquisition of low-abundance bacteria and revealed higher and more complex polymicrobial communities than previously reported (Ping et al., 2015). In our study, approximately 1467 OTUs belonging to 31 phyla and 557 genera were detected, and the majority of low-abundance bacteria were obtained by Illumina sequencing.

At the phylum level, the predominant bacterial phyla detected were *Proteobacteria, Firmicutes, Bacteroidetes*, and *Actinobacteria*. Consistent with our study, *Proteobacteria* and *Firmicutes* and *Bacteroidetes* were previously reported, by next-generation sequencing, as the most dominant phyla in primary and persistent infections (Siqueira et al., 2011; Hong et al., 2013; Keskin et al., 2017; Sánchez-Sanhueza et al., 2018; Hou et al., 2021). At the genus level, *Fusobacterium* with a mean relative abundance of 9.8% is the most abundant genus, which is consistent with previous studies (Siqueira et al., 2011; Tzanetakis et al., 2015). Pereira et al. (2017) used real-time PCR to test ten important microorganisms collected from the root apex and the surrounding apical lesion. *Fusobacterium nucleatum* was the most prevalent and significant species in the root canal and periradicular lesions (Pereira et al., 2017; Gomes et al., 2021). Meanwhile, Noguchi et al. (2005) found that *F. nucleatum* was frequently detected in extraradicular biofilm attached to the surface of root ends with persistent infection. Thus, *Fusobacterium* may be significantly related to the persistent infection.

In the present study, *Streptococcus* was frequently and dominantly detected in both intraradicular and extraradicular infections, which is in accordance with previous studies (Siqueira et al., 2002; Rocas et al., 2008; Takahama et al., 2018). *Streptococcus* was one of the most abundant and prevalent genera before and after the chemo-mechanical procedure (Zandi et al., 2018). Thus, we speculate that *Streptococcus* can survive in harsh nutritional conditions and play important role in persistent infection.

The PCoA based on the bray-curtis distances showed that intraradicular infection, extraradicular biofilm, and matched periapical lesion samples had similar microbiota profiles. Similar microbial profiles from different sites were previously demonstrated by comparing the bacterial composition from different researches (Noguchi et al., 2005; Kuremoto et al., 2014).

The sinus tract, which is the channel connecting the oral cavity and periapical lesions, was reported to occur one in five teeth with periapical lesions (Gupta and Hasselgren, 2003). The formation of the sinus tract is related to bacterial infection. It is still unclear whether there are bacteria that determine the formation of the sinus tract, or whether the formation of the sinus tract will cause the change of microbiome. In a previous study, fifty endodontic pathogens were detected using a closed-ended reverse-capture checkerboard method, and none of the taxa were significantly associated with the sinus tract (Rocas et al., 2011). However, Qi et al. (2016) found that taxa were significantly associated with the sinus tract targeting 11 species using microarrays. In the present study, the PCoA and PERMANOVA analysis revealed some differences in the bacterial composition of the infections with or without sinus tracts. The occurrence of sinus tracts may relate to the microbial communities of extraradicular infections associated with persistent apical periodontitis.

In the present study, *Porphyromonas*, *Eubacterium*, *Treponema*, *Phocaeicola*, *Tannerella*, and *Prevotella* were more associated with infections with the sinus tract. *Porphyromonas*, a genus of small anaerobic gram-negative nonmotile cocci, play an essential role in endodontic infections in light of its prominent virulence factors (Barbosa-Ribeiro et al., 2021; Bordagaray et al., 2021; Gomes et al., 2021) The sinus tract may be related to the simultaneous occurrences of both *Porphyromonas endodontalis* and *Tannerella forsythia*, *P. gingivalis*, and *P. endodontalis*, or *Parvimonas micra* and *P. endodontalis* (Qi et al., 2016). Bacterial communities of primary intraradicular infections with or without sinus tracts were compared, and a higher abundance of *P. gingivalis* and *F. nucleatum* sp. were associated with sinus tracts (Sassone et al., 2008). Previous studies concluded that *Tannerella* and *Treponema* were associated with periodontitis, with a higher presence in deep periodontal pockets (Farias et al., 2012). On the one hand, the occurrence of bacteria related to periodontitis may be the reason for the formation of the sinus tract. And on the other hand, the appearance of the sinus tracts may allow bacteria in the oral cavity to invade the area of persistent periapical infection.

The samples without sinus tracts were characterized by *Microbacterium* and *Enterococcus*. *Enterococcus*, a genus of gram-positive, facultatively anaerobic bacteria, was much more likely to be found in cases of persistent infections than in primary infections due to its ability to invade the dentinal tubules and highly resistant to antimicrobial strategies (Sun et al., 2009; Murad et al., 2014; Keskin et al., 2017; Bouillaguet et al., 2018; Sun et al., 2018; Barbosa-Ribeiro et al., 2021; Gomes et al., 2021; Zhang et al., 2021). In the present study, *Enterococcus* was consistently detected in all of the samples but with a low mean proportion of OTUs. Similar to our study, *Enterococcus* was detected as a low-abundant (0.7%) genus of persistent endodontic infections (Hong et al., 2013) and the core status of *Enterococcus* as the main pathogen of endodontic failures need to be further investigated.

## Conclusions

In conclusion, our study using high-throughput sequencing offers a detailed characterization of the intraradicular and matched extraradicular microbiome in persistent infections. And it confirms the polymicrobial nature of persistent endodontic infections. Diverse bacteria were detected in both intraradicular and extraradicular infections associated with persistent apical periodontitis. The occurrence of the sinus tract may relate to the extraradicular bacterial communities. Further endodontic microbiome studies are warranted to focus on bacterial pathogenicity and characterize correlations of microbial communities.

## Data Availability Statement

The datasets presented in this study can be found in online repositories. The names of the repository/repositories and accession number(s) can be found in the article/supplementary material.

## Ethics Statement

The studies involving human participants were reviewed and approved by Ethics Committee of the Dental School of the Capital Medical University, Beijing, China. The patients/participants provided their written informed consent to participate in this study.

## Author Contributions

XS, YN, and BH designed the research. ZY performed the experiment and analysis, XS and YN wrote the manuscript. All authors contributed to the article and approved the submitted version.

## Funding

This work was supported by Science and Technology Planning Project of Beijing Municipal Science & Technology Commission (Z191100006619037), Beijing Natural Science Foundation (7202058), and Scientific Research Common Program of Beijing Municipal Commission of Education (KM201810025026).

## Conflict of Interest

The authors declare that the research was conducted in the absence of any commercial or financial relationships that could be construed as a potential conflict of interest.

## Publisher’s Note

All claims expressed in this article are solely those of the authors and do not necessarily represent those of their affiliated organizations, or those of the publisher, the editors and the reviewers. Any product that may be evaluated in this article, or claim that may be made by its manufacturer, is not guaranteed or endorsed by the publisher.
